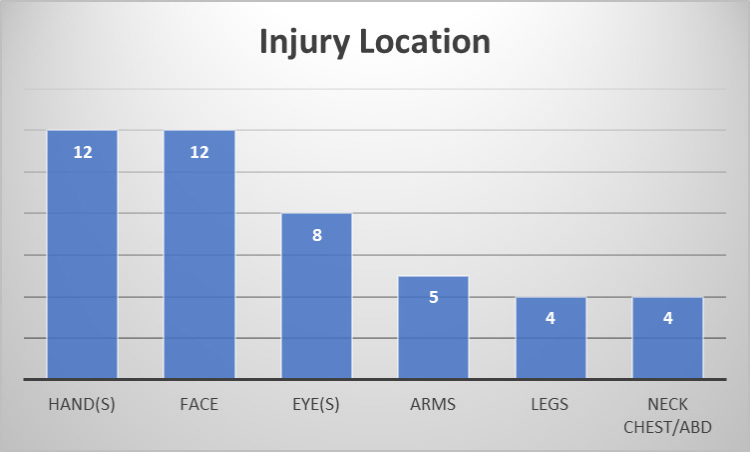# 536 Firework Injuries in Pediatric Patients: A 15 Year Review

**DOI:** 10.1093/jbcr/irad045.133

**Published:** 2023-05-15

**Authors:** Mark Johnston, Heidi Altamirano, Sam Miotke

**Affiliations:** Regions Hospital, Saint Paul, Minnesota; Regions Hospital, Saint Paul, Minnesota; Regions Hospital, Saint Paul, Minnesota; Regions Hospital, Saint Paul, Minnesota; Regions Hospital, Saint Paul, Minnesota; Regions Hospital, Saint Paul, Minnesota; Regions Hospital, Saint Paul, Minnesota; Regions Hospital, Saint Paul, Minnesota; Regions Hospital, Saint Paul, Minnesota

## Abstract

**Introduction:**

The United States Consumer Product Safety Commission (CPSC) estimated that 15,600 firework-related injuries were treated in US hospital emergency departments during calendar year 2020 and that there has been a trend of increased injuries from 2005-2020. The goal of this project was to look at the experience of one center with a large referral network, to identify trends and opportunities.

**Methods:**

A retrospective chart review was performed to analyze data points including mechanism, age, sex, TBSA, injury locations, procedures performed, outcomes and complications length of stay, and services involved. The burn registry was utilized to identify cases throughout a 15-year period.

**Results:**

During this period, 24 pediatric patients required admission for firework injuries. Eighty-four percent were males compared to 16% in females. Most injuries (67%) occurred while the patient was holding the firework and lighting it as opposed to being struck by a firework. Most of the injuries had small TBSA percentages (84% < TBSA of 15). In addition, most were referred from outside facilities. Most common sites of injury included the hands and face, followed by the eyes. Sixty percent of patients required operative intervention for their injuries, some requiring multiple surgeries. The average length of stay was 2 days, while 8 patients stayed longer than 5 days. Ninety-two percent were admitted to the burn service with additional specialty consultants. Most children required multiple follow up visits.

**Conclusions:**

Firework related injuries in children result in significant injury often requiring surgical procedures along with a long outpatient treatment course. This work highlights a need for continued advocacy, education, an intervention. This program uses a curriculum and process for children who are injured due to setting fires. The program is evaluating a similar model of intervention and 1:1 counseling for children injured due to fireworks.

**Applicability of Research to Practice:**

This project is applicable to practice as it highlights the continued opportunity for intervention and prevention for firework related injuries. The trends are not decreasing, and the injuries are significant. Evaluation of a model for youth who have fireworks related injuries, similar to the youth fire-setting program, would be a beneficial next step.